# Effect of Peri-Interventional Blood Loss on In-Stent Thrombosis After Percutaneous Coronary Intervention in Patients with Acute Myocardial Infarction

**DOI:** 10.3390/jcdd12020067

**Published:** 2025-02-12

**Authors:** Akram Youssef, Ahmed Mashaly, Usama Alkomi, Marian Christoph, Ahmed Abdelsamad, Silvio Quick, Nesma Elzanaty, Adrian Mahlmann, Karim Ibrahim, Tamer Ghazy

**Affiliations:** 1 Department of Internal Medicine and Cardiology, Klinikum Chemnitz, Flemmingstrasse 2, 09116 Chemnitz, Germany; akram.youssef@skc.de (A.Y.); usama.alkomi@skc.de (U.A.); m.christoph@skc.de (M.C.); s.quick@skc.de (S.Q.); karim.ibrahim@skc.de (K.I.); 2 Department of Cardiology, Tanta Faculty of Medicine, Tanta University, El-Bahr Street, Tanta 31111, Egypt; 3 Cardiovascular Center, Korea University Guro Hospital, Seoul 08308, Republic of Korea; 4 Department of General Surgery, Evangelisches Krankenhaus Lippstadt (EVK)-Hospital, 59555 Lippstadt, Germany; ahmed.abdelsamad@klinikum-vest.de; 5 Department of Medical Physiology, Tanta Faculty of Medicine, Tanta University, El-Bahr Street, Tanta 31111, Egypt; nesma.elzanaty@med.tanta.edu.eg; 6 Vascular Center South Westphalia, Clinic of Angiology, St.-Josefs Hospital, Katholisches Krankenhaus Hagen, 58097 Hagen, Germany; 7 Department of Internal Medicine, University Hospital, Ruhr University Bochum, 44801 Bochum, Germany; 8 Department of Cardiac Surgery, Marburg University Hospital, Philipps University of Marburg, Baldingerstrasse, 35043 Marburg, Germany

**Keywords:** peri-interventional bleeding, acute myocardial infarction, in-stent stenosis

## Abstract

This paper evaluates the effect of blood loss on in-stent stenosis after percutaneous coronary intervention (PCI) in patients with acute myocardial infarction (AMI). Nine hundred and ninety-seven patients who underwent PCI for AMI as well as follow-up coronary angiography at 6–12 months from two centers were categorized into three groups based on peri-interventional blood loss at the primary intervention (mild, <1 mmol/L moderate, 1–2 mmol/L; severe > 2 mmol/L). The endpoint was to evaluate the incidence and severity of in-stent stenosis at follow-up angiography and the revascularization rate. The incidence of in-stent stenosis and revascularization in mild, moderate, and severe groups was 19.3%, 33.1%, and 61.1%, respectively (*p* = 0.001), with HR: 1.35 (95% CI; 1.10–1.65), *p* < 0.001. Peri-interventional blood loss was associated with a higher incidence of in-stent stenosis and revascularization 6–12 months after successful PCI in patients with AMI.

## 1. Introduction

Coronary artery disease (CAD) is a serious cardiovascular disease worldwide and a leading cause of death in both developed and developing countries [1]. Major bleeding is the most frequent non-ischemic complication in patients with acute coronary syndrome (ACS) managed with percutaneous coronary intervention (PCI). In a recent cohort of patients with ACS, major bleeding events occurred in approximately 10% of the patients [2]. Patients with major bleeding also showed a 60% increased risk of in-hospital death and a fivefold increase in one-year mortality. Moreover, one-year myocardial infarction (MI) rates are almost five times higher in ACS patients with major bleeding [3].

In contrast, in-stent stenosis is a well-known complication of PCI, with well-identified risk factors [4,5,6,7,8]. However, the role of peri-interventional blood loss in in-stent stenosis remains unclear. This study aimed to determine the impact of peri-interventional blood loss on the incidence and severity of in-stent stenosis and target vessel revascularization, as detected by routine coronary angiography 6–12 months after primary PCI.

## 2. Patients and Methods

### 2.1. Study Design

This was a retrospective observational cohort study of patients successfully treated with primary PCI for AMI (including ST-elevation and non-ST-elevation myocardial infarctions) at the Cardiovascular Center of Korea University Guro Hospital (KUGH), Seoul, Republic of Korea, and the Department of Cardiology, Dresden Heart Center, Dresden Technical University, Dresden, Germany. All procedures were performed after obtaining adequate patient consent, and the review boards of both institutions approved the study protocol.

Hemoglobin levels were measured at admission and discharge.

Acute myocardial infarction was defined as the detection of an increase and/or decrease in cardiac troponin levels with at least one value above the 99th percentile of the upper reference limit (URL), together with evidence of ischemia. Ischemia was defined as any symptom of ischemia, electrocardiographic (ECG) changes suggestive of new ischemia (new ST elevation, left bundle branch block [LBBB], the development of pathological Q waves), or imaging evidence of infarction. Established MI was defined as any criterion that satisfied the development of new pathologic Q waves on serial ECGs, imaging evidence of MI, or pathologic findings of healed or healing MI [9].

In routine coronary angiography, in-stent stenosis is considered angiographically significant if it is >50% of the stented segment diameter, as assessed by quantitative coronary angiography.

### 2.2. Patient Population

All patients who underwent PCI for AMI between November 2004 and May 2014 were included. Of 5593 patients, 997 met the inclusion criteria of age above 18 years and routine coronary angiography 6–12 months after PCI.

The study population was categorized into three groups according to peri-interventional blood loss: mild (Hb loss < 1 mmol/L), moderate (Hb loss 1–2 mmol/L), and severe (Hb loss > 2 mmol/L).

### 2.3. Procedural Details

Experienced interventional cardiologists performed primary PCI and routing angiography. The culprit lesion was identified based on its morphology, including complete occlusion, thromboembolism, and ulcerative stenosis, or if these features were absent, it was assumed to be the tightest stenosis. Simple predilatation was performed to attain an adequate luminal diameter necessary to accommodate the unexpanded drug-eluting stents (DES) and their delivery system. Thrombus aspiration or mechanical thrombectomy was performed only when clinically indicated.

### 2.4. Medical Treatment

All patients received a dual antiplatelet regimen composed of a loading dose of aspirin (200–300 mg) followed by life-long administration of 100 mg/d, as well as a loading dose of clopidogrel (300–600 mg) followed by 75 mg/d for at least 12 months. Cilostazol (100 mg loading, followed by 100 mg twice/d for at least one month) was added as an adjunct to the standard dual antiplatelet therapy in high-risk patients at the physician’s discretion.

Antithrombotic therapy for PCI was performed using a therapeutic dose of enoxaparin before PCI till the patient was discharged, and a reduced dose of unfractionated heparin (bolus of 50–70 U/kg). Glycoprotein IIb/IIIa blockers were administered at the discretion of the physician.

### 2.5. Statistical Analysis

All statistical analyses were performed with SPSS software (version 20.0; IMB Inc., Chicago, IL, USA). Continuous variables were expressed as mean ± standard deviation, and statistical comparisons were made by one-way analysis of variance (ANOVA). Categorical data were expressed as percentages and compared using chi-square statistics or Fisher’s exact test. A *p*-value of less than 0.05 was considered statistically significant.

Event rates of clinical endpoints were estimated by the Kaplan–Meier analysis and Cox proportional hazards regression analysis, as hazard ratios and 95% confidence intervals were estimated, and a probability value of 0.05 or less was considered statistically significant. Multivariate logistic regression, which included baseline confounding factors, was used to assess the precise and independent impact of peri-interventional blood loss on the study endpoints. We tested all available variables of potential relevance, including age, sex, and cardiovascular risk factors (hypertension, diabetes, dyslipidemia, current smoking and alcohol consumption, multi-vessel disease, stent type, post-dilation, and dissection).

## 3. Results

### 3.1. Patient Demographics

Patients with severe peri-interventional Hb loss (>2 mmol/L) included more females, had lower left ventricular ejection fraction, and had higher chronic kidney disease rates than those with mild or moderate peri-interventional Hb loss (<2 mmol/L). Other risk factors did not appear to affect peri-interventional blood loss (Table 1). None of the patients received blood transfusion during hospital stay.

Angiographically, patients with severe peri-interventional Hb loss had significantly higher multi-vessel coronary disease, higher number of treated vessels, left main disease, higher rate of bare metal stent (BMS) implantation, and minimal need for post-dilation than those with mild and moderate peri-interventional Hb loss (Table 2).

### 3.2. Clinical Outcomes

**Figure 1:**
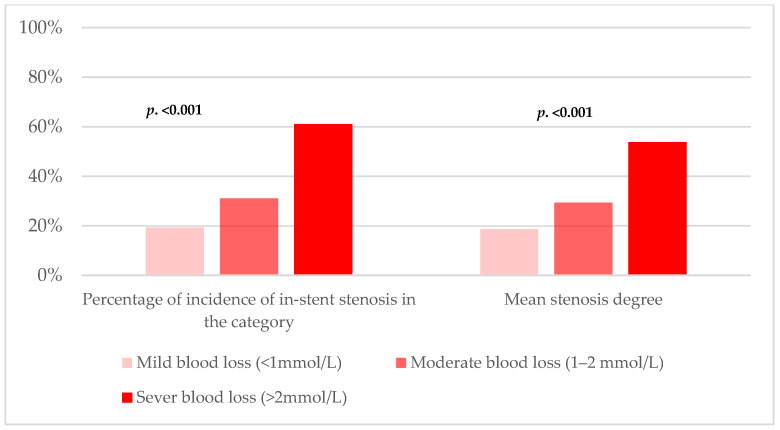
Rate of in-stent Stenosis and degree of stenosis by degree of blood.

The rate of in-stent stenosis was significantly higher in the patients with severe peri-interventional Hb loss compared to the patients with moderate and mild Hb loss (61.1%, 33.1%, and 19.3%, respectively; log-rank < 0.0001, with HR: 1.35; 95% CI: 1.10–1.65, *p* < 0.001). Regarding the severity of the stenoses, the mean stenosis was 53.8 ± 34.7% in the severe group compared to 29.4 ± 37.2% and 18.7 ± 32.6% in the moderate and mild group, respectively (*p* < 0.001), Figure 1.

### 3.3. Predictors of Angiographically-Detected In-Stent Stenosis

Univariate logistic regression analysis identified peri-interventional blood loss, anemia, older age, lower left ventricular ejection fraction, diabetes mellitus, dyslipidemia, chronic kidney disease, smoking, alcoholism, multi-vessel disease, left main disease, BMS implantation, and coronary dissection as significant variables affecting the incidence of in-stent stenosis (ISS) and target vessel revascularization (TVR). In the multivariate logistic regression model describing factors independently associated with an increase in the ISS and TVR, peri-interventional blood loss was an independent predictor of ISS in our population ([HR: 1.612; 95% CI: 1.25–2.06, *p* < 0.001, and HR: 1.17; 95% CI: 1.04–1.31, *p* = 0.008] respectively), Table 3.

## 4. Discussion

Blood loss after PCI is a common complication requiring transfusion in up to 8.9% of all interventions [10] and is associated with higher morbidity and mortality at the primary intervention; however, it does not include short-term coronary reocclusion [2,11].

Denes et al. [12] demonstrated increased myointimal cell proliferation after hypoxia and reoxygenation in a carotid model [12]. A similar effect could be responsible for coronary in-stent stenosis after intervention, in which low hemoglobin levels exaggerate hypoxia. This could indicate a correlation between periprocedural blood loss and in-stent stenosis during follow-up. This study investigated the effect of periprocedural blood loss on midterm stent patency.

Similar to previous observations, the population of patients with anemia in this study was older and had a higher prevalence of diabetes [13,14,15]. The prevalence of anemia in our population was approximately 30%, higher than the global average [16]. This could be explained by the fact that anemia can exacerbate myocardial ischemia and precipitate acute coronary syndrome through reduced tissue oxygen delivery. Anemia is also associated with particular clinical and biological profiles, including high blood pressure, renal failure, malnutrition, subclinical atherosclerosis, and lower limb arteritis. This profile is frequently observed in patients with coronary artery disease [17].

Similar to the general coronary patient demographic profile, more men than women were in the patient cohort. Interestingly, this difference was not observed in the severe hemoglobin loss group. Another statistically significant characteristic between the severe Hb loss group and the other two groups was reduced left ventricular function. Both female sex and reduced ventricular function, albeit variably, are associated with in-stent stenosis [18,19]. This suggests synergism between these factors. The same applies to the higher prevalence of bare metal stents in the severe hemoglobin loss group.

Acikgöz et al. [20] found a relationship between general stenosis tendencies and stent stenosis. In our patient cohort, the group with severe Hb loss had significantly more peripheral arterial disease with platelet aggregation inhibitors, which may have contributed to a higher bleeding tendency and more severe periprocedural blood loss. However, this did not appear to protect against stent stenosis in our cohort.

This study found that peri-interventional blood loss in patients with AMI undergoing PCI, independent of transfusion, impacted the rate and severity of ISS 6–12 months after the intervention. To our knowledge, this is the first study to investigate this relationship. We found this correlation dependent on the degree of blood loss, with a higher rate of stent stenosis and more severe involvement at a blood loss > 2 mmol/L. This seems to be related to the complexity of the intervention rather than the target vessel, as shown by more blood loss and stent stenosis occurring in patients with more target vessels and left main disease, less angiographic success, and less clinical success. In contrast, there was no significant difference in revascularized vessels.

Interestingly, there was also a statistically significant difference in the type of stent implanted, with more bare-metal stents used in the group with greater blood loss and thrombosis. Although some studies have demonstrated a higher rate of stent thrombosis, others have not reported such findings, even in non-coronary settings. Other studies have shown similar differences during long-term follow-up [21,22,23,24,25]. In this regard, the higher rate of stent stenosis in the severe Hb loss group could be attributed to the increased use of bare-metal stents; however, this effect should be further investigated.

A similar question also arises regarding using a post-dilatation balloon, regarding which the literature demonstrates a split opinion [26,27,28,29]. The number of post-dilatation balloons was significantly lower in the severe Hb loss group, which may or may not have affected the rate of in-stent stenosis.

It is widely recognized that the morphology and type of coronary stenosis play a crucial role in determining the course and success of an intervention, as well as the risk of potential complications. In our study, we did not collect direct data on the classification of lesion types or detailed lesion morphology. However, data were collected and analyzed that indirectly allow inferences about these parameters. A central aspect of our analysis was the investigation of the necessity of using an NC balloon (non-compliant balloon) for lesion preparation. The use of an NC balloon indicates the presence of a highly calcified lesion. This suggests that patients requiring an NC balloon had morphologically complex and calcified lesions. In addition, it was documented whether thrombus aspiration was necessary before stent implantation. This procedure also provides insights into lesion morphology, particularly regarding the presence of thrombotic components. Furthermore, we recorded the exact diameters of the implanted stents for each patient. These data allow for conclusions about the size of the treated lesions. Longer stents and stents with larger diameters were used for extensive lesions, making the dimensions and complexity of the lesions indirectly discernible. Another important aspect was the assessment of the re-restenosis rate following stent implantation with in-stent restenosis and its treatment. An increased re-restenosis rate could indicate complex lesions with a predisposition for recurrent in-stent restenosis. To assess the complexity of the intervention, we documented the fluoroscopy time for each patient. A longer fluoroscopy time can be interpreted as an indicator of technically demanding and complex interventions. Finally, the angiographic success of each intervention and the occurrence of peri-interventional complications were recorded. These parameters provide important information about the immediate success of the treatment and the risks associated with the treatment of specific lesion types. Our findings demonstrate that, although direct assessment of lesion morphology and lesion type was not performed, indirect parameters such as the use of an NC balloon, the necessity of thrombus aspiration, stent size, restenosis rate, and fluoroscopy time offer valuable insights into the complexity and morphology of the treated lesions. These data allow for important conclusions to be drawn regarding the course and outcome of the intervention.

Based on the presented results, we recommend maintaining good hemostasis, which is more pronounced in the PCI of patients with multi-vessel disease and those requiring complex interventions. We also recommend aggressive management of periprocedural anemia and blood loss, especially in patients with blood loss of >2 mmol/L. Moreover, we endorse radial artery access, which has been proven to minimize site-related bleeding events [30,31].

Nevertheless, these recommendations were based not only on the presented study results, but also on published data reporting increased all-cause mortality in patients treated with DES, as well as increased cardiac mortality and an increased risk of myocardial infarction in the presence of anemia. Stortecky and colleagues reported an increased incidence of stent thrombosis in the presence of severe anemia (5.1% in patients with severe anemia compared to 2.3% in patients with mild or no anemia) [32]. A study by Hong YJ et al. investigated the influence of anemia on coronary plaque in patients with acute coronary syndrome (ACS). The volumetric analysis revealed that patients in the anemia group had a larger plaque volume and a smaller lumen volume compared to the non-anemia group. In addition, there was a negative correlation between hemoglobin levels and the volume of the necrotic nucleus [33]. Thus, periprocedural bleeding and the resulting anemia contribute to the increase in MACE in patients with ACS after PCI regardless of the other risk factors. Potential mechanisms such as anemia-induced intimal hyperplasia, inflammatory cytokine activation, and impaired oxygen transport might be responsible [33].

In our study, the patients with anemia tended to be older and had a higher prevalence of diabetes mellitus as well as arterial hypertension. The well-known cardiovascular risk factors that can lead to atherosclerosis are also risk factors for in-stent restenosis. As described above, anemia and periprocedural bleeding increase the rate of MACE. In our patient group, anemia occurred more frequently in patients with diabetes mellitus than in those without this disease (36.1% vs. 27.9%). According to a study by J. Barbieri et al., the prevalence of anemia was in 34.2% of patients with type 2 diabetes [34]. A direct relationship between anemia and arterial hypertension has not been explicitly described in the literature. However, iron deficiency is known to be a common cause of anemia and is often found in patients with heart failure [35]. The Framingham Heart Study identified arterial hypertension as one of the main factors that may contribute to the development of heart failure. These findings could explain the more frequent anemia findings in patients with arterial hypertension.

Despite the development of DES and the advancement of PCI technology, in-stent restenosis continues to occur in patients after PCI [36]. Several well-known factors that lead to the development of in-stent restenosis have already been mentioned in our work. It is clear that the rate of in-stent restenosis has been significantly reduced by the development of DES in recent years, but it is still being reported [37]. If the hypothesis of peri-interventional Hb decline increasing the rate of in-stent restenosis is further confirmed in future studies, it may, and even should, have further implications on future, not only short-term, but also mid-term target hemoglobin levels after PCI.

The focus of the study was to evaluate if blood loss per se has an effect on the in-stent restenosis. Further research might re-visit the topic, evaluating the now-standard transradial approach and its effect on blood loss, and hence in-stent restenosis. Nevertheless, evaluating the different approaches’ effects should come as the second step after reporting the blood loss as a relevant factor.

### Study Limitations

This was an observational, retrospective cohort study. Moreover, we only included asymptomatic patients undergoing routine follow-up coronary artery angiography; however, there is still a chance of selection bias in selecting high-risk patients or patients with complex coronary morphology for routine follow-up coronary angiography, which may explain the relatively high stenosis rates in our cohort compared to those in previous reports. Furthermore, LDL and HbA1C levels were not measured for all patients in the follow-up and were at the discretion of the family doctor.

Based on this unavoidable bias, the data results should be interpreted with caution and the results might be more applicable in higher-risk patients.

The blood loss categorization was at the discretion of investigators. We also could not perform propensity score matching analysis of the three patient groups due to the relatively small sample size of patients with severe Hb loss; however, we performed univariate and multivariate logistic regression analyses to identify the impact of peri-interventional Hb loss as an independent predictor of risk factors. Therefore, larger randomized trials with stronger statistical power must confirm these findings.

## 5. Conclusions

Our data are hypothesis-generating results highlighting the increased risk of in-stent stenosis in patients with peri-interventional blood loss, which is more common in those with severe blood loss. Therefore, large randomized trials are required to confirm these patients’ increased risk of restenosis.

## Figures and Tables

**Table 1:** Baseline characteristics.

	<1 mmol/L	1–2 mmol/L	>2 mmol/L	*p*-Value
Variables, N (%)	(*n* = 694)	(*n* = 151)	(*n* = 36)	
Sex, male	545 (78.5)	113 (74.8)	19 (52.8)	**0.001**
Age, year	61 ± 11.6	61.6 ± 10.3	61.3 ± 14.5	0.840
Initial hemoglobin (mmol/L)	8.6 ± 0.9	8.7 ± 1.0	8.5 ± 1.1	0.543
LV ejection Fraction, %	48.7 ± 10.3	47.5 ± 11.5	43.4 ± 11	**0.009**
STEMI	360 (51.9)	84 (55.6)	20 (55.6)	0.661
NSTEMI	334 (48.1)	67 (44.4)	16 (44.4)	0.661
History of coronary artery disease	50 (7.2)	12 (7.9)	1 (2.8)	0.553
Hypertension	357 (51.4)	72 (47.7)	19 (52.8)	0.685
Diabetes mellitus	217 (31.3)	45 (29.8)	13 (36.1)	0.762
Dyslipidemia	258 (37.2)	61 (40.4)	22 (61.1)	**0.014**
history of PAD	11 (1.6)	2 (1.3)	2 (5.6)	0.185
history of CKD	102 (14.7)	38 (25.2)	14 (38.9)	**0.001**
Smoking history	343 (49.4)	64 (42.4)	18 (50)	0.285
Current	270 (38.9)	56 (37.1)	16 (44.4)	0.714
Alcoholic history	193 (27.8)	32 (21.2)	3 (8.3)	**0.012**

**CKD:** chronic kidney disease; **LV:** left ventricle; **PAD:** peripheral arterial disease; **STEMI:** ST-segment elevation myocardial infarction; **NSTEMI:** non-ST-segment elevation myocardial infarction. The bold numbers are the significant values.

**Table 2:** Angiographic data.

	Hemoglobin Loss			*p*-Value
Variables, N (%)	<1 mmol/L	1–2 mmol/L	>2 mmol/L	
	(*n* = 694)	(*n* = 151)	(*n* = 36)	
Muli-vessel disease	266 (38.3)	81 (53.6)	23 (63.9)	**0.001**
No. of vessels	1.5 ± 0.7	1.8 ± 0.86	1.9 ± 0.82	**<0.001**
Target vessel				
LM	12 (1.7)	8 (5.3)	3 (8.3)	**0.004**
LAD	361 (52)	82 (54.3)	21 (58.3)	0.689
LCX	190 (27.4)	38 (25.2)	10 (27.8)	0.853
RCA	261 (37.6)	61 (40.4)	13 (36.1)	0.791
RAMUS	9 (1.3)	1 (0.7)	1 (2.8)	0.572
BMS	138 (19.9)	49 (32.5)	16 (44.4)	**<0.001**
DES	558 (80.4)	104 (68.9)	20 (55.6)	**<0.001**
Post-dilatation balloon	309 (44.5)	45 (29.8)	9 (25)	**0.001**
Dissection	48 (6.9)	22 (14.6)	2 (5.6)	**0.007**
Angiographic success	691 (99.6)	151 (100)	35 (97.2)	**0.082**
Clinical success	693 (99.9)	151 (100)	35 (97.2)	**0.004**

**BMS:** bare-metal stent; **DES:** drug-eluting stent; **LAD:** left anterior descending artery, **LCX:** left circumflex artery; **LM:** left main coronary artery; **RCA:** right coronary artery.

**Table 3:** Full univariate and multivariate Cox proportional hazards models for predictors of target vessel revascularization.

	Univariate		*p*-Value	Multivariate		*p*-Value
	HR	95% CI		HR	95% CI	
Anemia	1.57	1.21–2.04	0.001	1.612	1.25–2.06	**<0.001**
HB loss	1.19	1.07–1.33	0.001	1.17	1.04–1.31	**0.008**
Age	0.985	0.97–0.997	0.014	0.992	0.97–1.0	0.299
LV ejection Fraction, %	0.985	0.97–0.99	0.012	0.986	0.97–1.0	**0.043**
Diabetes mellitus	1.32	1.02–1.71	0.035	1.071	0.79–1.44	0.650
Dyslipidemia	1.32	1.02–1.71	0.029	1.026	0.72–1.46	0.885
history of CKD	1.4	1.08–1.82	0.011	1.259	0.88–1.80	0.208
Smoking history	1.33	1.02–1.72	0.030	0.997	0.54–1.83	0.993
Current	1.37	1.04–1.81	0.022	0.784	0.42–1.45	0.438
Alcoholic history	1.43	1.01–2.03	0.033	0.989	0.61–1.59	0.965
Multi-vessel disease	1.4	1.08–1.82	0.009	1.014	0.56–1.82	0.962
No. of vessels	1.23	1.06–1.43	0.006	1.069	0.75–1.50	0.704
LM	3.24	1.84–5.69	<0.001	3.254	1.74–6.08	**<0.001**
BMS	1.38	1.07–1.79	0.011	0.526	0.06–4.22	0.545
DES	0.72	0.56–0.093	0.014	0.463	0.05–3.78	0.472
Post-dilatation balloon	0.74	0.58–1.04	0.050	0.889	0.58–1.34	0.574
Dissection	1.64	1.05–2.57	0.029	1.863	1.14–3.04	0.013

**BMS:** bare metal stents, **CI:** confidence interval; **CKD:** chronic kidney disease; **DES:** drug-eluting stents; **HB:** hemoglobin; **HR:** hazard ratio; **LDL:** low-density lipoprotein; **LM:** left main; **LV:** left ventricular; **STEMI:** ST-segment elevation myocardial infarction; **NSTEMI:** non-ST-segment elevation myocardial infarction.

## Data Availability

The data supporting the findings of this study are available from the first author upon reasonable request. The data will be available for five years after publication.
